# Comparative genomics and genome biology of *Campylobacter showae*

**DOI:** 10.1080/22221751.2019.1622455

**Published:** 2019-06-06

**Authors:** Tiffany Hsu, Matthew R. Gemmell, Eric A. Franzosa, Susan Berry, Indrani Mukhopadhya, Richard Hansen, Monia Michaud, Hans Nielsen, William G. Miller, Henrik Nielsen, Mona Bajaj-Elliott, Curtis Huttenhower, Wendy S. Garrett, Georgina L. Hold

**Affiliations:** a Department of Biostatistics, Harvard T. H. Chan School of Public Health, Boston, USA; b School of Medicine, Medical Sciences and Nutrition, Centre for Genome Enabled Biology and Medicine, University of Aberdeen, Aberdeen, UK; c School of Medicine, Medical Sciences and Nutrition, GI Research Group, University of Aberdeen, Aberdeen, UK; d Department of Paediatric Gastroenterology, Royal Hospital for Children, Glasgow, UK; e Departments of Genetics and Complex Diseases and Immunology and Infectious Diseases, Harvard T. H. Chan School of Public Health, Boston, USA; f Department of Clinical Microbiology, Aalborg University Hospital, Aalborg, Denmark; g Produce Safety and Microbiology Research Unit, U.S. Department of Agriculture, Agricultural Research Service, Albany, USA; h Department of Infectious Diseases, Aalborg University HospitalAalborg, Denmark; i Infection, Immunity, Inflammation Programme, UCL Great Ormond Street Institute of Child Health, London, UK; j St George and Sutherland Clinical School, Microbiome Research Centre, University of New South Wales, Sydney, Australia

**Keywords:** *Campylobacter showae*, comparative genomics, genome biology, gastrointestinal disease, bacterial virulence factors, bacterial secretion systems

## Abstract

*Campylobacter showae* a bacterium historically linked to gingivitis and periodontitis, has recently been associated with inflammatory bowel disease and colorectal cancer. Our aim was to generate genome sequences for new clinical *C. showae* strains and identify functional properties explaining their pathogenic potential. Eight *C. showae* genomes were assessed, four strains isolated from inflamed gut tissues from paediatric Crohn’s disease patients, three strains from colonic adenomas, and one from a gastroenteritis patient stool. Genome assemblies were analyzed alongside the only 3 deposited *C. showae* genomes. The pangenome from these 11 strains consisted of 4686 unique protein families, and the core genome size was estimated at 1050 ± 15 genes with each new genome contributing an additional 206 ± 16 genes. Functional assays indicated that colonic strains segregated into 2 groups: adherent/invasive vs. non-adherent/non-invasive strains. The former possessed Type IV secretion machinery and S-layer proteins, while the latter contained Cas genes and other CRISPR associated proteins. Comparison of gene profiles with strains in Human Microbiome Project metagenomes showed that gut-derived isolates share genes specific to tongue dorsum and supragingival plaque counterparts. Our findings indicate that *C. showae* strains are phenotypically and genetically diverse and suggest that secretion systems may play an important role in virulence potential.

## Introduction

Historically, *Campylobacter jejuni* and *Campylobacter coli* have been recognized as intestinal pathogens that contribute to significant health and economic costs [1,2]. Recent studies have also found that other *Campylobacter* species, termed non-jejuni/coli (njc)-campylobacters or “emerging *Campylobacter* species”, are associated with a range of diseases, including gastroenteritis, periodontitis, inflammatory bowel disease (IBD), and cancer [3–5]. The prevalence of human illness associated with emerging *Campylobacter* species is currently lower than for *C. jejuni* and *C. coli-*associated illness and are thought to be heavily underreported [6,7]. One such *njc-Campylobacter* is *Campylobacter showae*, a commensal of the human oral cavity long linked with periodontal disease [8,9]. It has recently been associated with inflammatory bowel disease [4, 10–12] and colorectal cancer [5].

*C. showae* originally formed part of the *Campylobacter rectus* group until it was formally reclassified by Etoh et al. in 1993 [8]. *C. showae* are flagellated, H_2_S-producing Gram-negative rods that can grow in microaerophilic or anaerobic conditions but have a requirement for fumarate and formate or hydrogen. To date, little work has been done to characterise pathogenicity markers in *C. showae*. Three genomes currently exist: a closed genome for oral isolate ATCC 51146, also known as RM3277, along with two draft genome sequences from colonic tissue isolates. CSUNSWCD was isolated from a paediatric Crohn’s disease patient mucosal biopsy [13], while CC57c came from a colonic tumour specimen [5]. Genomic analyses have indicated that the genomes of these two colonic strains are diverged from ATCC 51146 [5,13] and contain virulence factors that are not detected in this oral strain. Both CSUNSWCD and CC57c contain components of a Type IV secretion system, with CSUNSWCD also shown to contain phage proteins that were not present in the oral strain. However, both studies were limited to single strain comparisons to oral strain ATCC 51146 and, to date, no comparative genomic analyses have been performed with multiple *C. showae* strains.

In this study, we profile the pathogenicity and functionality of 11 *C. showae* strains using both whole genome comparisons and experimental approaches. The strains encompass the three published reference strains and eight newly-isolated strains from four patients and were subjected to a combination of Illumina and PacBio sequencing. We characterized the eight new strains through experimental assays to assess their ability to adhere to, invade, and infect host cells and monitored their response to host colonic tissue explants. We defined the pan- and accessory genomes of *C. showae* using whole genome comparisons and searched for differences between strains that were adherent/invasive versus those that were not. Lastly, we describe differences between oral and colonic strains by comparing gene family presence/absence between our *C. showae* isolates and *C. showae* within oral metagenomes from the Human Microbiome Project [14]. Considering their association with a number of chronic inflammatory diseases, it is important to understand the pathogenic mechanisms used by *C. showae* within the gut to define their impact on human health.

## Materials and methods

### Bacterial isolates

All bacterial isolates used in the study were obtained from patient samples. Information on sample types and disease presentation are shown in Table 1. All patients gave informed consent, and ethical approval was obtained from the local ethics committees.

**Table 1.** C. showae strains characterized in this study.

Strain	Patient	Patient type	Sample type	Diagnosis	Country of origin	Sequencing Platform^	Adhesion to colonic epithelial cells^+^	Invasion of colonic epithelial cells*
B32_SW	B32	Paediatric	Biopsy	Crohn’s	UK	IM + PB	4.85 ± 8.08	0.17 ± 0.03
B91_SC	B91	Paediatric	Biopsy	Crohn’s	UK	IM + PB	0.15 ± 0.68	0.00 ± 0.00
B91_SCBr	B91	Paediatric	Biopsy	Crohn’s	UK	IM	0.50 ± 1.08	0.00 ± 0.00
B91_TPP	B91	Paediatric	Biopsy	Crohn’s	UK	IM	0.27 ± 0.86	0.00 ± 0.00
129_MSG	129	Adult	Biopsy	Adenoma	UK	IM + PB	3.42 ± 8.12	0.10 ± 0.01
129_VTPP	129	Adult	Biopsy	Adenoma	UK	IM	4.68 ± 9.78	0.13 ± 0.03
129_VTPPs	129	Adult	Biopsy	Adenoma	UK	IM	2.45 ± 3.04	0.11 ± 0.01
CAM	CAM	Adult	Stool	Gastroenteritis	Denmark	PB	1.34 ± 2.72	0.33 ± 0.07

Note: All strains’ isolation location was colonic, and all strains were positive in the Vero cell line cytotoxicity assay. Results for adhesion and invasion assays are mean ± SD of independent experiments.

^IM (Illumina MiSeq), PB (PacBio-RSII).

^+^Mean number of bacteria ± SD per HT-29 cell after 6 h incubation.

*Mean percentage ± SD of the original inoculum after 1 h gentamicin treatment of 6h-infected HT-29 cells.

### Functional assays

#### Adherence and invasion

Unless otherwise stated, all bacterial cultures were grown in Columbia broth (CB) supplemented with 0.2% sodium formate, 0.3% sodium fumarate, and 10% Fetal Calf Serum (FCS) in an anaerobic incubator at 37°C for 3 days. We assessed the ability of all strains to adhere to and invade intestinal epithelial cell lines by conducting gentamicin protection assays with HT-29 epithelial cells (American Type Culture Collection (ATCC)). We followed the methods of Man et al. [15] for epithelial cell studies including scanning electron microscopy, except that we used HT-29 cells instead of Caco-2 cells.

#### Bacterial cytotoxicity assay

Bacterial cytotoxicity was assayed by adding bacterial supernatant extracts to Vero (African green monkey kidney) tissue culture monolayers. Bacterial supernatant extracts were prepared by centrifugation (13 000 X g, 5 min) of overnight cultures grown in CB supplemented with 0.2% sodium formate, 0.3% sodium fumarate, and 10% FCS. Supernatants were filtered through 0.2 μm membrane filters (Millipore Corporation, Bedford, MA). Vero cell monolayers were prepared in 96-well microtitre plates (Corning, USA) with Dulbecco’s Modification of Eagle’s Medium (DMEM) containing L-glutamine (Gibco Ltd) and 5% Fetal Bovine Serum (FBS), with incubation in 5% CO_2_ at 37°C for 24 h. Supernatant filtrates were added to Vero cells and incubated for 2 days at 5% CO_2_ at 37°C; the negative control was phosphate-buffered saline (PBS). Cells were assessed for cytotoxicity by looking for rounding of cells.

#### Galleria mellonella killing assays

*G. mellonella* larvae (Live Foods) were infected with *C. showae* strains in 50 μL inocula (10^6^ CFU of each *C. showae* strain) by microinjection in the right foreleg. The larvae were incubated at 37°C, and survival and appearance were recorded at 24 h. PBS, media-injected (CB supplemented with 0.2% sodium formate, 0.3% sodium fumarate, and 10% FCS) and un-injected controls were also used. Survival 24 h after infection was recorded. For each experiment, ten *G. mellonella* larvae were infected, and experiments were repeated three times.

#### Host cytokine secretion

Cytokine secretion of tumour necrosis factor alpha (TNF-α) and interleukins IL-8 and IL-10 from co-culture supernatants derived from the human monocytic leukemia cell line (THP-1; ATCC) was measured as previously described [16] using a custom-designed Human Magnetic Luminex Screening Assay (R&D Systems, MN, USA) (Supplementary text).

#### Explant studies

Murine colonic explants were prepared from eight to twelve-week-old wild-type C57BL/6 mice bred in house and originally purchased from Jackson Laboratory. The colon was resected and flushed with sterile PBS. The tissue was opened longitudinally and washed repeatedly with sterile PBS to eliminate the remaining intestinal content and immediately placed in DMEM with 10% FCS. The tissues were divided into 5-mm-long sections under sterile conditions and incubated at 37°C in humidified 5% CO_2_ atmosphere. After 10 min, the media was replaced, and the samples were incubated for an additional 2 h in the same medium with or without 10^6^ cfu/ml of the various *C. showae* strains (grown in Columbia broth (CB) supplemented with 0.2% sodium formate, 0.3% sodium fumarate, and 10% Fetal Calf Serum (FCS) in an anaerobic incubator at 37°C for 3 days), before being harvested and diluted in the DMEM with 10% FCS and then either snap-frozen in liquid nitrogen for RNA extraction or fixed overnight in 4% paraformaldehyde, followed by routine paraffin embedding, sectioning and staining with H&E.

#### Biofilm analysis

Biofilm formation was examined during the stationary culture *in vitro* in 24-well polystyrene microplates (24 F-Well Microplates, Thermo Fisher Scientific™ Nunc™, Denmark) using a 0.1% crystal violet (CV) stain as previously described [17]. The media used was CB supplemented with 0.2% sodium formate, 0.3% sodium fumarate, and 10% FCS (Supplementary Text).

### Expression analysis

Total RNA from bacterial strains and colonic explant studies were isolated using the RNeasy kit (Qiagen). cDNA was prepared from 1 µg of RNA using the Iscript cDNA synthesis kit (Bio-Rad). Quantitative PCR (qPCR) was done on a Stratagene MX3005P instrument using the KAPA SYBR Green PCR Kit (Kapa Biosystems). The qPCR primers and efficiencies are shown in Supplementary Table 1. Relative expression data were generated using the ddCt method, and corrected for primer efficiencies according to Pfaffl et al. [18]. Relative expressions from intestinal explants and bacterial samples were normalized against the relative expression of the housekeeping gene glucose-6-isomerase. This was done using the formula Expression_(gene of interest)_/Expression_(housekeeping gene)_ = relative expression.

### Genome sequencing

We performed short-read sequencing for all eight strains, of which four (B32_SW, B91_SC, CAM & 129_MSG) were also subjected to long-read sequencing. For sequencing (long and short-read), genomic DNA was extracted from *C. showae* liquid cultures as described previously [19]. Information on *de novo* assembly (genomes and plasmids) and gene annotation can be found in the Supplementary Text file.

### Comparative genomics approaches

#### Determining phylogenetic relationships

A phylogenetic tree based on the assembled genomes of the *Campylobacter showae* strains was produced using REference ALignment based PHYlogenetic builder (REALPHY) and RAxML (Supplementary Text). The *Campylobacter rectus* RM3267 genome was used as an outgroup for the phylogenetic analysis. REALPHY aligns genomes to a reference genome/s and uses aligned sets of orthologous sites (SNPs as well as nonpolymorphic sites) to reconstruct phylogeny. The number of SNPs detected for each *C. showae* strain were similar ranging from 64,703 to 68,264. This analysis was repeated with the strains CSUNSWCD and B32_SW excluded.

Gubbins was utilized to detect levels of recombination and to produce phylogenetic tree whilst excluding areas of recombination [20]. This analysis requires a reference genome; ATCC 51146 was used as a reference as it was the most contiguous and complete genome.

BRIG (BLAST Ring Image Generator) was utilized to create a graphical representation of the similarities of genomic content within the *C. showae* genom*es* [21]. This requires one genome to be used a reference to the rest; B32_SW_DW was utilized as the reference, as it was the largest assembly. Assembled plasmid contigs were BLASTed against each other with BLASTN to detect similar plasmids across samples.

We used the CLARK sequence classification method (v1.2.3) with a custom database to classify the strains B91_SC, B91_SCBr and B91_TPP. CLARK runs were carried out for each strain using a kmer size of 31 (default). The genomes used within the custom database included multiple *Campylobacter* genome assemblies (see Supplementary Text).

#### Pangenome generation and exponential decay calculation

Pan- and accessory genomes were generated for assembled and reference genomes. We first used usearch cluster_fast [22] to cluster all *Campylobacter* proteins from the CLARK custom database above (*n* = 27 902), using a 90% amino acid similarity cut-off, in order of decreasing length. This resulted in 4686 protein families for our 11 isolates and reference strains. Protein families were then aligned via DIAMOND against the HUMAnN2-formatted UniRef90/UniRef50 databases [23]. Only protein families that aligned with ≥90% similarity and ≥80% coverage to a UniRef90 were annotated (*n* = 3 514). To determine which protein families were plasmid-derived, we used DIAMOND to align the protein families against plasmid proteins (which were called by running Prokka on the plasmid assemblies). Protein families that aligned to a plasmid protein with ≥90% similarity and ≥90% coverage were annotated as plasmid-derived (for the strain the plasmid was from). Of the 4 686 protein families, 524 were from plasmids.

We next estimated the size of the core genome and calculated the number of new protein families added per genome. To do this, we generated two datasets. For the first dataset, we determined the number of shared protein families between *n* (1 through 11) genomes in up to 100 combinations, while for the second dataset, we computed the number of new protein families with the addition of the *nth* genome in up to 100 combinations. We fitted each dataset to the exponential decay equation, *F*(*n*) = *κ* × exp(−n/τ) + Ω as outlined in Tettelin et al. [24]. For the first dataset, Ω estimates the size of the core genome, while for the second dataset, Ω estimates the number of new genes added per genome. The “nls” function in R was used to fit each dataset to the above equation using starter values from their paper (*κ* = 610, *τ* = 2.16, Ω = 1806). We then estimated the number of genes in the pangenome using the equation, *F*(*n*) = *D *+ (Ω × (n−1)) + (*κ* × exp(−2/τ)) * ((1−exp(−(n−1)/τ))/(1−exp(−1/*τ*))), in which *D* is the average number of genes per sequenced genome, and *κ*, *τ*, and Ω are estimated from the second dataset (*κ* = 8201.0209, *τ* = 0.6778, Ω = 206.0316).

#### Assessing virulence factors and antibiotic resistance genes

The Virulence Factor Database (VFDB) and Comprehensive Antibiotic Resistance Database (CARD) were used to detect virulence factors and antibiotic resistance genes in the assembled and annotated chromosomes and plasmids (both downloaded September 2016) [25,26]. This was carried out by performing both nucleotide-nucleotide and protein-protein alignments (see Supplementary Text for detail).

#### Comparison to oral metagenomes

We acquired HUMAnN2 (http://huttenhower.sph.harvard.edu/humann2) gene family profiles for metagenomes from the Human Microbiome Project I Phase II Project (HMP1-II) [27]. We then filtered for gene family profiles in which *C. showae* recruited reads to (1) ≥500 UniRef90 gene families [28], with (2) reasonably strong coverage (median non-zero abundance ≥10 reads per kilobase (RPK), and 3) consistent coverage (3rd quartile / 1st quartile of non-zero abundances <2.5). This resulted in 64 metagenomes from the supragingival plaque (*n* = 43), tongue dorsum (*n* = 18), and buccal mucosa (*n* = 3), respectively. HUMAnN2 *C. showae-*specific gene families are derived from a *C. showae* pangenome constructed from isolate genomes RM3277, CSUNSWCD, and CC57c within MetaPhlAn2 [29]; genes were clustered at 97% similarity with UCLUST [22]. To compare our 11 *C. showae* isolate genomes to the oral metagenomes, we aligned each isolate gene to the MetaPhlAn2-generated pangenome and annotated each gene with a pangenome element. This allows for standardization of gene families across metagenomes and isolate genomes.

A file containing the R markdown script for bioinformatics analysis and figure generation is supplied in the Supplementary Information.

## Results

### Sequencing and assembly of *C. showae* genomes

Eight clinical *C. showae* strains were obtained from four patients (Table 1). From two children with newly diagnosed but treatment naive Crohn’s disease we isolated four strains: a single *C. showae* isolate (B32_SW) from one patient, and three *C. showae* strains (B91_SC, B91_SCBr and B91_TPP) with differing colony morphology from the second patient. All isolates were obtained from inflamed colonic mucosa [30]. The remaining four strains were isolated from adults. A single isolate (CAM) was from a patient with gastroenteritis and three *C. showae* strains (129_MSG, 129_VTPP and 129_VTPPs) with differing morphology were obtained from a patient with a colonic adenoma. CAM was isolated from stool while the latter three isolates were from the adenoma.

We sequenced seven isolates using Illumina short-read sequencing and, for the B91_SC, 129_MSG, CAM and B32_SW isolates, also performed PacBio RSII long-read sequencing (see Methods). In these cases, where both the short and long reads were of sufficient quality (B91_SC, 129_MSG and B32_SW), we scaffolded short-read assemblies with PacBio subreads to generate more complete assemblies (including plasmids; see below). The assembled genome for CAM was derived purely from PacBio long-read sequencing. Assemblies contained both chromosomal and plasmid content; plasmids were separately called and analyzed later (see below). Quality and completeness of assembled genomes were estimated, alongside the publicly-available genomes ATCC 51146 (reference oral strain), CSUNSWCD and CC57c (colonic isolates), using QUAST, BUSCO, and REAPR [31–33], indicating high contiguity, high completeness, and low error for all new genomes (Supplementary Figure 1; Supplementary Table 2 and 3). In each of the resulting assemblies, only the publicly available CC57c and CSUNSWCD genomes were demonstrated to have low levels of completeness. The longest contig for the CC57c and CSUNSWCD assemblies made up less than 25% of the assembly length, while the longest contig within all of the new genomes was >57.8% of the assembly length (Supplementary Figure 1). All genomes were estimated to be >98% complete by BUSCO, except CC57c and CSUNSWCD with completeness estimates of 92.9% and 97.6% respectively (Supplementary Figure 1; Supplementary Table 2). All new genome assemblies had 6 or fewer contigs, while the existing published colonic strains, CC57c and CSUNSWCD had 274 and 23 contigs respectively. Genome sizes ranged from 2.1 Mb to 2.6 Mb with 38–131x Illumina read depth (Supplementary Table 4). One of the new genome assemblies (B91_SC), which had been subjected to short and long-read sequencing, yielded a single contig containing all assembled genome information. The genomes were analyzed to determine if there was a presence of indels introduced through sequencing error to a specific strain. Our isolates showed very similar results to each other except for 129_VTPP which had more prodigal annotated proteins; 1389 compared to the range of 1150–1243 for the other isolates (Supplementary Table 5). The publically available samples (CC57, ATCC 51146, and CSUNSWCD) were also similar but of slightly lower quality.

### Genome analysis identifies heterogeneity in phylogenetic relationships and genome structure between *C. showae* strains

**Figure 1.**
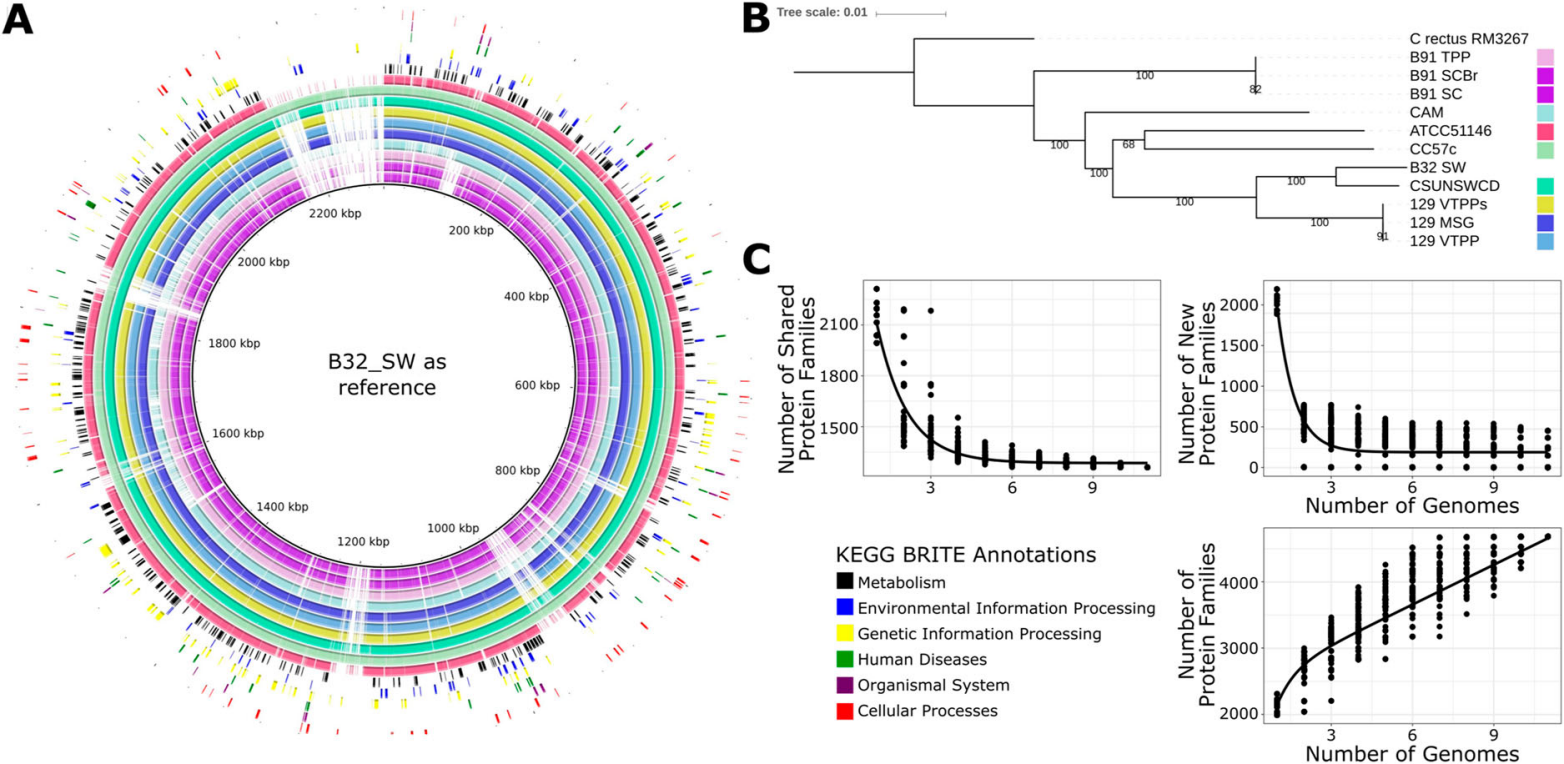
Genome assemblies of eight newly-characterized *C. showae* strains. (A) BRIG (BLAST Ring Image Generator) [21] showing the assembled genomes aligned to the longest genome (B32_SW). KEGG BRITE [58] functional categories are annotated on the image to indicate where they are located within the genome, and strains are denoted by colors shown on the (B) phylogenetic tree (whole genome alignment, generalized time reversible model; see Methods). Bootstrap values are shown at each node. (C) Estimated number of core, new, and pan-genes within the *C. showae* clade with fits calculated using an exponential decay model.

The genomes of the 11 *C. showae* strains were compared using BRIG (Figure 1(A)), and a phylogenetic analysis was performed using whole-genome-sequence alignments with the *C. rectus* RM3267 genome as the outgroup (Figure 1(B)). The three *C. showae* strains isolated from patient 129 were almost identical (Figure 1(B)). Isolate B32_SW appeared most closely related to the paediatric colonic strain, CSUNSWCD, with these five strains forming a clade. In contrast, CAM did not cluster particularly with any other *C. showae* strains. Its closest relatives were the oral strain ATCC 51146 and the colonic tumour isolate CC57c. The three strains from paediatric patient B91 are almost identical but did not form a monophyletic clade with the other *C. showae* strains and were most closely related to *C. rectus*. To confirm whether the B91 strains were indeed *C. showae,* we used CLARK to classify the genome assemblies with a custom database of *Campylobacter* species genomes. All the B91 strains were classified as *C. showae*, with only very small scaffolds (<1500 bp and one scaffold of B91_TPP (12,505 bp)) being classified as other *Campylobacter* species or not at all (Supplementary Table 6).

Homologous recombination plays a large role for adaptation for *C. jejuni* [34]. We assessed the extent of horizontal sequence transfer within the *C. showae* strains using the ATCC 51146 strain as a reference. A phylogenetic tree excluding predicted regions of horizontal gene transfer was produced with the level of recombination across the genomes shown (Supplementary Figure 2A). This phylogeny was highly similar to that of the whole genome (Figure 1(B)), with two main differences. First, CC57c and ATCC 51146 do not form a monophyletic clade, and second, CAM and the B91 strains (B91_SC, B91_SCBr & B91_TPP) did not form a monophyletic clade in the recombination excluded phylogeny.

The degree of predicted recombination varies widely across the *C. showae* strains; however, the predicted recombination regions are shared within the (1) 129 clade and within the (2) B91 and CAM clade. The highest levels of recombination were detected in the strains B32_SW and CSUNSWCD, with 225 and 170 recombination blocks, respectively, spanning >500,000 bases each (Supplementary Table 7). In contrast, the recombination areas in all other strains comprised <100,000 bases. The 129 strains (129_VTPP, 129_VTPPs & 129_MSG) contained 74,770–78,938 bases in recombination areas, of which the vast majority was shared between the three strains. ATCC 51146 & CC57c contained 10,961 & 15,975 bases in recombination areas, respectively. The B91 strains (B91_SC, B91_SCBr & B91_TPP) & CAM contained the lowest levels of recombination with 4388–4584 bases in recombination, which were also largely shared.

Phylogeny reconstruction of the whole genomes with REALPHY was repeated without CSUNSWCD and B32 SW (Supplementary Figure 2B), the two strains with the highest recombination, which resulted in a tree highly similar to the original (Figure 1(B)) Like the phylogeny without recombination (Supplementary Figure 2(B)), ATCC 51146 and CC57c did not form a monophyletic clade.

**Figure 2.**
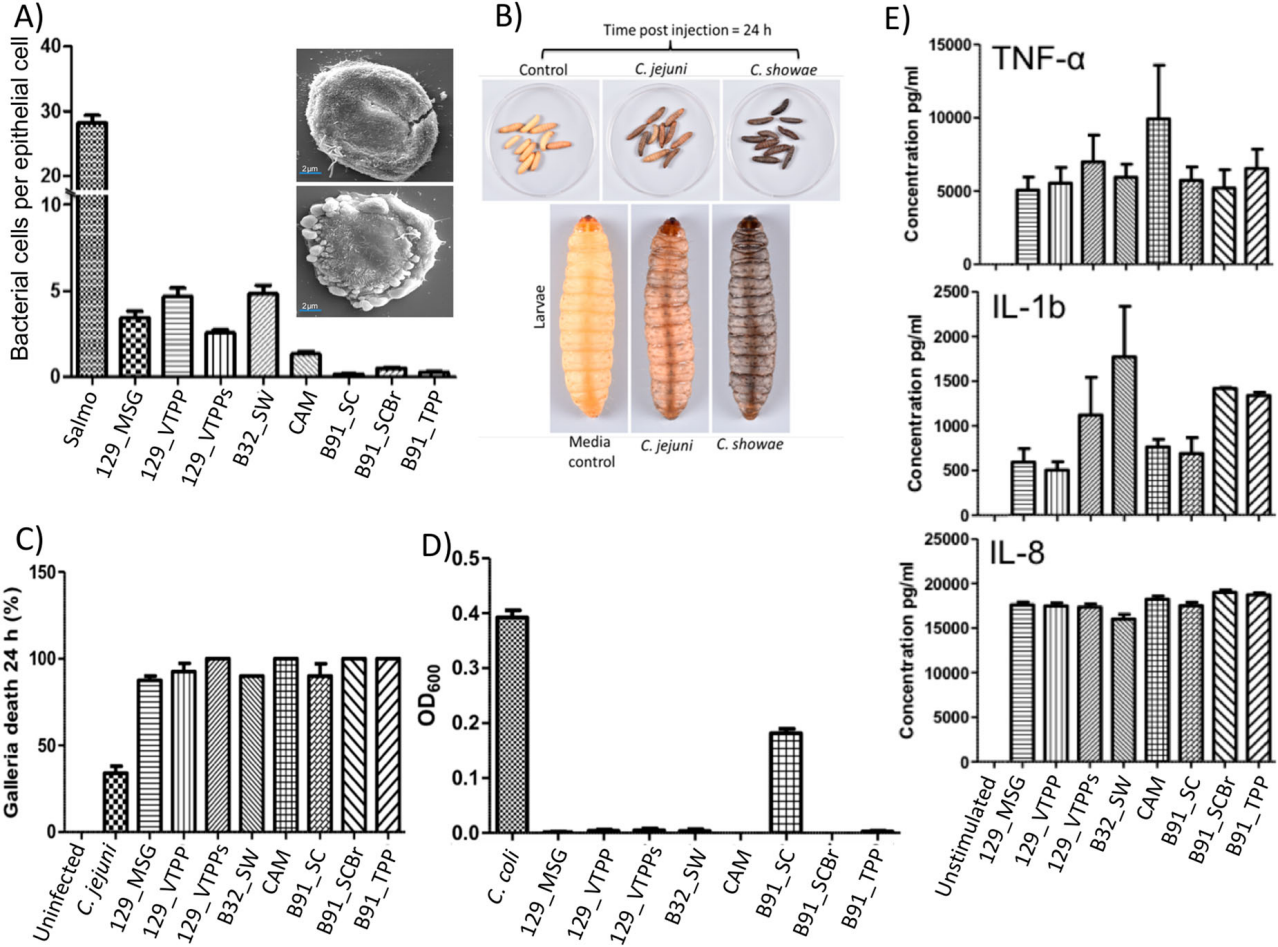
Colonic *C. showae* strains demonstrate a range of virulence potential. (A) Adhesion to HT29 colonic epithelial cells with SEM. For images within inset: B32_SW- (top) and CAM-infected (bottom) cells. (B) and (C) *Galleria mellonella* killing. Groups of 10 larvae were used and the results shown are the means of three experiments recorded 24 h after challenge. Error bars indicate standard errors of the mean. *P < 0.05 (survival compared with *C. jejuni*; Welch-corrected 2-tailed *t* tests for pooled data from three separate experiments). (D) Evidence of biofilm formation in isolate B91_SC. Results shown are the means of three independent experiments recorded 24 h after inoculation. Error bars indicate standard errors of the mean. (E) Cytokine production from THP1-cells. Error bars indicate standard errors of the mean.

We next quantified the size of the *C. showae* pangenome within these new isolates and reference genomes. To do so, we annotated the genomes using Prokka, clustered protein sequences at 90% amino acid similarity, and annotated the resulting families with UniRef90 identifiers. Of the 4 686 protein families, the core genome (protein families shared by all isolates) comprised 1 260 families, the accessory genome (protein families shared between two or more strains) included 1 881 families, and unique proteins (protein families unique to 1 strain) consisted of 1 545 families (Supplementary Figure 3, Supplemental Table 8). Core proteins included DNA polymerases I and III, along with known *Campylobacter* species markers, such as the flagellar genes *flgG, flhA, flhF, flhB, fliP, fliR* and *fliS*. *C. showae* requires fumarate and formate for growth [8], and we observed genes for fumarate metabolism (e.g. genes encoding the three subunit fumarate reductase FrdABC) and genes for formate metabolism (formate dehydrogenase, formate hydrogenlyase, and formate-tetrahydrofolate ligase) (Supplemental Table 8). 98.96% of the unique protein families belonged to five strains: CC57c (*n* = 387), CAM (*n* = 478), B32_SW (*n* = 265), ATCC 51146 (*n* = 244) and CSUNSWCD (*n* = 155) (Supplementary Figure 3). The six remaining genomes had genes encoding seven or fewer unique proteins, reflecting the high degree of similarity within the three strains from patient B91 and the three strains from patient 129. Using an exponential decay function [24,35], we estimated the *C. showae* core genome to be 1 284 ± 7 genes and that each new genome added an additional 200 ± 14 genes. For comparison, *Streptococcus agalactiae* and *Escherichia coli* are estimated to have core genomes of 1 806 and 2 200 genes, respectively, and relatively large pangenomes (each genome adds approximately 33 and 300 genes, respectively) (Figure 1(C)).

Using IslandViewer [36], we detected significant genomic islands within all genomes, suggesting that the *C. showae* genome has undergone horizontal gene transfer (Supplemental Table 9). We were able to identify a number of predicted genomic islands in each genome and performed PCR validation of several prophage clusters, which confirmed the presence of the various transposases and integrases.

### Functional assessment of virulence and pathogenic potential of *C. showae* strains

Since these strains were isolated from patients, we profiled strain characteristics such as the ability to (i) adhere to and invade epithelial cells (Table 1), (ii) secrete virulence factors, and (iii) infect larvae of the greater wax moth *Galleria mellonella*. First, we observed that the adherence level of the *C. showae* strains to HT29 colonic epithelial cells ranged from 0.15 to 4.85 bacterial cells per epithelial cell (average of 100 epithelial cells counted per strain/per experiment; three independent experiments; Figure 2(A); Table 1). The three strains from patient B91 showed little evidence of adherence (average of 0.3 bacterial cells per epithelial cell), while the remaining five strains all showed adherence levels of >3.3 bacterial cells per epithelial cell (Table 1). The various adherent strains induced varying levels of cell damage (Figure 2(A)), with strain B32SW causing limited cell damage, whilst CAM strain caused extensive membrane blebbing and cell ultimately cell death. These five strains (129_MSG, 129_VTPP, 129_VTPPs, CAM, B32_SW) were also invasive, with invasion levels of HT-29 colonic epithelial cells ranging from 0.10% to 0.33% (Table 1), where a reference positive control strain of *Salmonella enterica* Serovar *typhi* had an invasion level of 20.3% ± 1.45% (data not shown). None of the strains could invade macrophages, although incubation of the *C. showae* strains with the THP-1 human monocytic leukemia cell line for 6 h led to proinflammatory cytokine production (TNF-α, IL-1b and IL-8) (Figure 2(E)). Second, we assessed the presence of secreted virulence factors by inoculating *C. showae* supernatants onto Vero cells. All culture supernatants demonstrated cytotoxic activity by increasing the size and rounding of Vero cells. Third, we assayed each strain’s ability to infect *Galleria mellonella* larvae*,* which are susceptible to infection by a number of bacterial pathogens including *C. jejuni* [37]. All larvae challenged with PBS or culture media and uninfected controls were alive after 24 h (Figure 2(B and C)). Larvae challenged with *C. jejuni* (10^6^ CFU) had approximately 70% survival; however, larvae challenged with *C. showae* (10^6^ CFU) had <10% survival. We also looked at whether *C. showae* strains were able to form biofilms, as several studies have shown that biofilms play a key role in bacterial colonization of the healthy gut and in intestinal diseases [38]. Bacteria growing in biofilms are also known to express increased virulence traits or increased resistance to antibiotics or host defense measures [39,40]. Strain B91_SC showed evidence of biofilm formation, which was not demonstrated by the other ten *C. showae* strains (Figure 2(D)).

**Figure 3.**
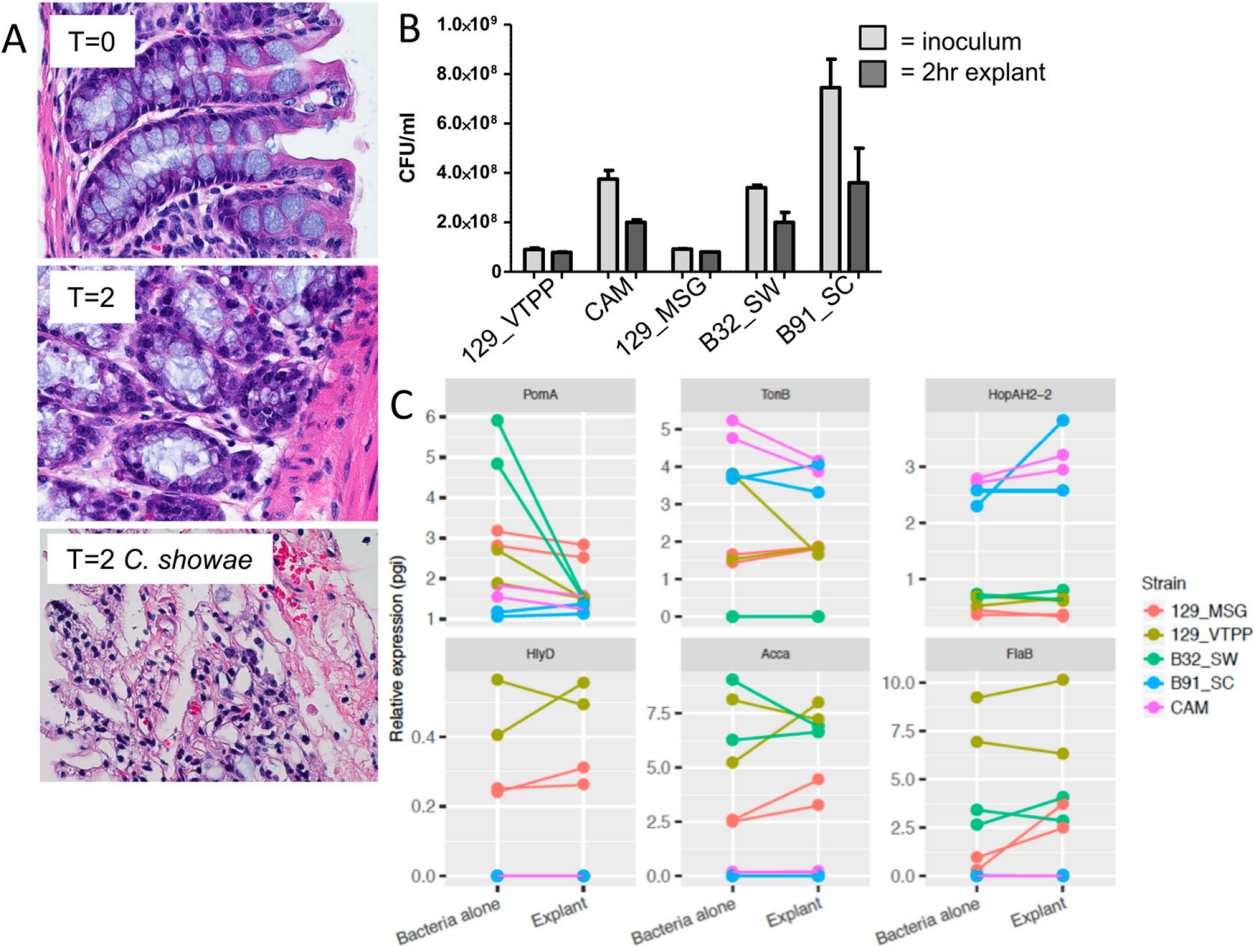
*Campylobacter showae* promotes loss of tissue architecture and exhibits altered pathogenicity in the host intestine. (A) Light micrograph of hematoxylin & eosin-stained formalin-fixed paraffin- embedded colonic tissue from C57BL/6J mouse colon explant co-cultures. (B) viability of *C. showae* strains following 2 hr explant co-culture. (C) RTqPCR analysis of a selection of *C. showae* virulence/pathogenicity genes in 2 hr pure culture and following 2 hr explant co-culture. Data are represented as mean ± SEM.

To understand how *C. showae* may behave within the gut, we cultured *C. showae* strains in the presence of murine large intestinal segments. Histology-based assessment indicated that colonic explants remained intact during the incubation period (2 hrs), however, *C. showae* strains caused dramatic loss of tissue architecture and integrity (Figure 3(A)). Microbiological analysis indicated that the *C. showae* cells remained viable during the incubation (on average ∼65% compared to initial counts, Figure 3(B)). All *C. showae* strains induced damage to colonic explant integrity. Real-time quantitative PCR (RTqPCR) analysis showed that *C. showae* virulence gene expression levels differ across strains and may change when in contact with colonic tissue. All strains except B91_SC had decreased expression of *pomA* (a motor protein involved in chemotaxis) when in contact with the colonic explant. 129_MSG, 129_VTPP, and B32_SW had higher expression of *hlyD* (involved in haemolysin secretion), *accA* (involved in lipid metabolism), and *flaB* (a non-essential flagellin protein) as compared to B91_SC and CAM (whether alone or with the colonic explant). In contrast, strains B91_SC and CAM had higher expression for *tonB* (involved in iron transport) and *hopAH-2* (part of the type III secretion system) relative to the other strains (whether alone or with the colonic explant). Strain 129_MSG showed increased expression for all three genes when inoculated with the colonic explant, while CAM showed decreased *tonB* expression and increased *hopAH-2* expression with the explant (Figure 3(C)).

The differential expression of these genes in the presence of the colonic explant may elucidate host protein alignments (see Supplementary Text for detail).

Comparison to oral pathogen interactions. Chemotaxis allows bacteria to swim towards or away from specific niches, and is utilized by a number of pathogenic bacteria, including *Helicobacter pylori* [41]. *pomA* expression in *C. showae* strains may decrease upon contact with the host as an energy conservation mechanism. The high expression of *flaB* in the adherent/invasive strains (129_VTPP, B32_SW, and 129_MSG) suggest an adjustment to growth conditions [42,43] or pathogenesis, as *C. jejuni* employs flagellar genes for secretion of non-flagellar proteins that modulate virulence [44]. Decreased expression of *tonB* along with upregulation of the type III secretion system may indicate how B91_SC and CAM derive metabolites from the host. This activation is in line with other Gram-negative bacterial systems, in which secretion systems (particularly Type III) are activated by bacterial contact with host cell surfaces [45]. CAM, an invasive strain, has similar gene expression patterns across all six genes when compared to B91_SC, a non-invasive and non-adherent strain. Together, these findings show that all of our *C. showae* strains are cytotoxic and infect *G. mellonella*; interestingly, the B91 strains are not adherent or invasive to epithelial cells, and strains vary widely in expression profiles when in contact with the host.

### Genomic differences between adherent/invasive *C. showae* isolate strains

**Figure 4.**
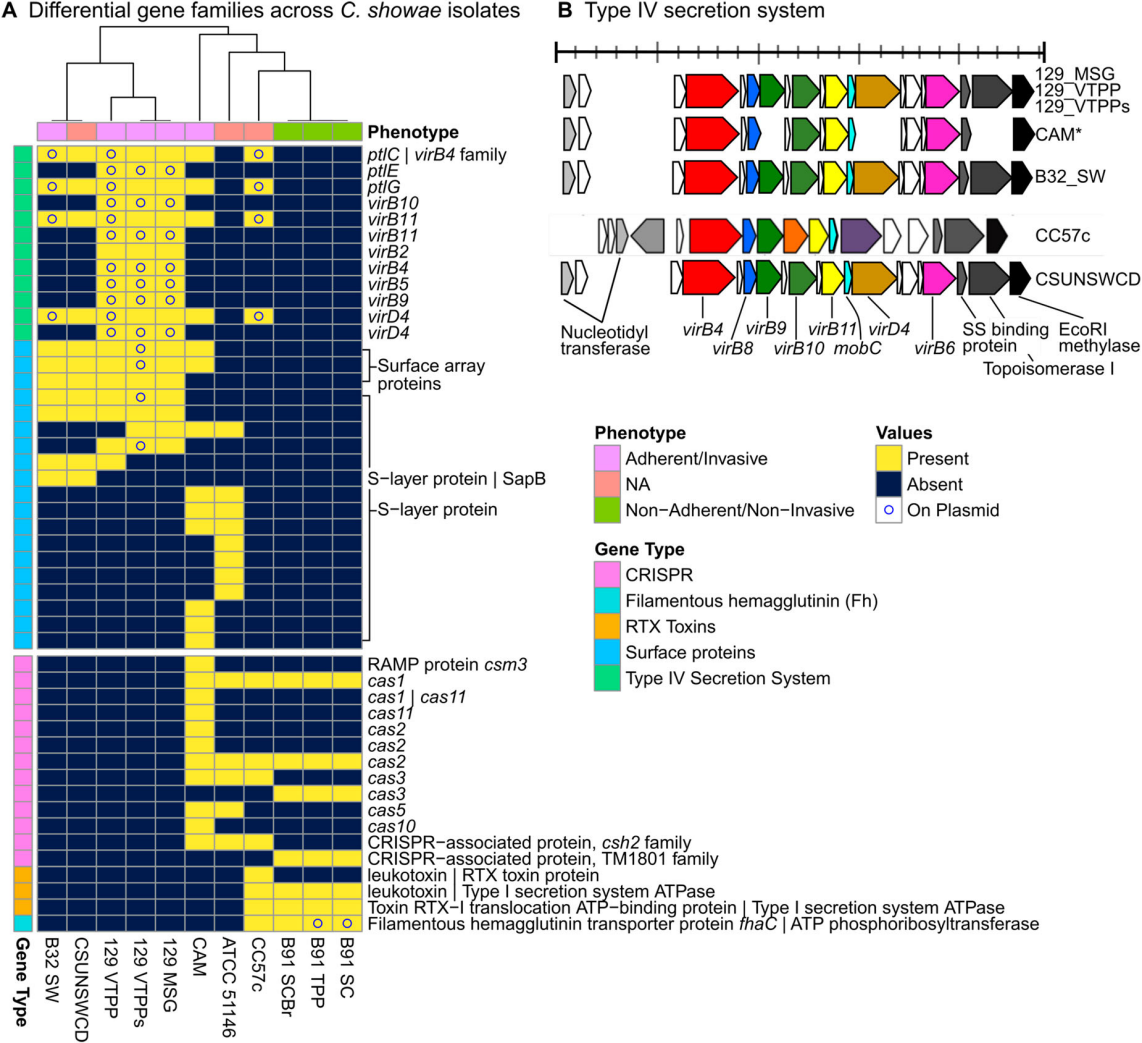
Gene families, including T4SS, virulence factors, and CRISPR-Cas systems, differ in adherent/invasive *C. showae* strains. (A) Gene families that differ in their presence/absence among *C. showae* isolates. A subset of genes of interest are labelled with Prokka and UniRef90 annotations when available. Isolates are clustered based on “Euclidean” distances using “complete” method. (B) We searched for Type IV secretion proteins in each *C. showae* genome, which are displayed here by position and direction based on Prokka annotations.

We next searched the pangenome for genetic and functional elements that might explain the differences between invasive/adherent strains (129_MSG, 129_VTPP, 129_VTPPs, CAM, B32_SW) and the non-invasive/non-adherent strains (those from patient B91) (Figure 4). The invasive/adherent strains contained Type IV secretion system (T4SS) and S-layer proteins, while non-invasive/non-adherent strains contained filamentous hemagglutinin, CRISPR, and repeats-in-toxin (RTX) proteins. We further assembled plasmids (Supplemental Table 10, Supplemental Table 11) and determined whether protein families were chromosomal or plasmid-borne. The presence of T4SSs exclusively in invasive/adherent strains clearly highlights differences in virulence traits between the strains. T4SSs generally mediate the contact-dependent transfer of DNA and protein substrates from bacteria to recipient hosts [46]. For example, in the *H. pylori* Cag-T4SS, host and environmental conditions (such as iron limitation) cause increased pili formation and translocation of the effector protein (*cagA*) into host cells via the T4SS [47]. For our isolates, all strains contained *pilZ* and 5 strains had *pilT*, which may be involved in type IV pilus assembly [48] or twitching mobility, respectively. The presence of pilus-related genes in *C. showae* is remarkable in itself, as its presence in *C. jejuni* has been controversial. Pili have been observed via electron microscopy but not tied to pili genes [49]. This observation of pili was refuted several years later [50] and subsequently regarded as an artifact attributable to the culture broth. We observed that multiple components of the T4SS were plasmid-derived for the strains from patient 129, B32_SW, and CC57c, but notably not CSUNSWCD.

Other proteins present only in the adherent/invasive strains included S-layer proteins, which again highlights the heterogeneity of virulence mechanisms exhibited by *C. showae.* S-layer proteins are capsule-like assemblages present on the cell surface of several Gram-negative pathogens including *Campylobacter fetus* and *C. rectus* [51]. They play a role in immune avoidance, in which they confer resistance to complement-mediated killing and cause down-regulation of pro-inflammatory cytokine levels. To date, in-depth analysis of S-layer protein composition of *C. fetus* and *C. rectus* strains has not been carried out, although it is recognized that S-layer composition in these pathogenic strains is subject to high frequency antigenic variation. In the *C. showae* analysis, four different S-layer protein families were found to be plasmid-derived for 129_VTPPs and are likely present on the chromosome in the other strains.

Proteins present only in the non-adherent/non-invasive strains included filamentous hemagglutinins, Cas and other CRISPR-associated proteins, and RTX toxins. None of these proteins were found on plasmids, other than the filamentous hemagglutinin transporter precursor protein FhaC. Filamentous hemagglutin has been used for adhesion in *Bordetella pertussis* but may also be used for iron acquisition [52]. CRISPR loci are found in certain bacteria where they confer resistance to exogenous genetic elements [53]. CRISPR-associated genes were found in the three strains from patient B91, CAM, CC57c, and ATCC 51146. They shared the CRISPR-associated endonuclease genes *cas1* and *cas2*, but only some strains encoded the helicase Cas3 and only the latter two strains encoded Cas5. To extend our understanding of CRISPR-Cas variation between isolates, we analyzed spacer content in the CRISPR positive strains using CRISPRCasFinder. We confirmed CRISPR-Cas systems in all of the above strains except CC57c, most being of type IB (Supplementary Figure 4; Supplementary Table 12). Absence of CRISPR-Cas systems has been associated with microbes that have obligate symbiotic lifestyles (as opposed to those with free-living lifestyles), since CRISPR arrays may increase the risk of acquiring spacers that target the host [54]. Lastly, the RTX toxins are virulence factors made by Gram-negative bacteria and may consist of either hemolysins or leukotoxins. In our study, hemolysins and leukotoxins were found only in the three strains from patient B91 and in CC57c. The presence of different toxins in adherent/invasive versus non-adherent/non-invasive strains may indicate varying virulence methods even within the same species.

### Virulence and antibiotic resistance profiling

We searched for virulence factors and antibiotic resistance genes by aligning all isolate proteins against the Virulence Factor Database (VFDB) (via BLASTP) and the Comprehensive Antibiotic Resistance Database (CARD) (via RGI) [25,26]. Our *C. showae* isolates aligned poorly to both reference databases, indicating that *C. showae* is distantly related generally to more-characterized pathogens (Supplementary Table 5). CARD identified 66 potential antibiotic resistance genes (ARGs) passing a 80% coverage threshold in C. jejuni, but only 5–9 ARGs among C. showae strains. Whilst our *C. showae* strains had 95–107 VFDB hits with >50 bitscore and 59–75 CARD hits with >50 bitscore the reference *C. jejuni* (NCTC 11168) had 157 VFDB hits with >50 bitscore and 1430 CARD hits with >50 bitscore. Furthermore, VFDB Our VFDB search against our *C. showae* strains and the *C. jejuni* reference detected a total of 170144 different virulence factors passing an 80% coverage threshold in C. jejuni, and 84–91 virulence factors in the C. showae strains 66 of these were only found only in the *C. jejuni* reference and 26 were not detected in the *C. jejuni* reference (Supplementary Figure 6).

Like *C. jejuni*, these proteins included 35 flagella-related proteins and 9 proteins related to pseudaminic acid synthesis. We also found adherence-related proteins, such as: the capsular proteins KpsF and CpsA; lipooligosaccharide proteins GmhA, GmhB, WaaC, HtrB and HldE; the invasion antigen CiaB (but not CiaC which requires a Type III secretion system); and the outer membrane fibronectin-binding protein CadF. The latter has been found to be essential for *C. jejuni* binding to INT 407 cells [55]. Some proteins distinguished between groups of strains: the adherent/invasive strains from patient 129 contained the HhuA haemoglobin-haptoglobin binding protein and shared the T4SS with CC57c, CSUNSWCD, B32_SW, and CAM, while the non-adherent/non-invasive B91 strains and CC57c carried capsule proteins Cap8O and Cps4l, that were not identified in the patient 129 strains. To determine which virulence factors were plasmid-derived, we aligned all plasmid proteins against VFDB (Supplementary Figure 6). We again detected the T4SS, as well as capsule genes *kpsF* and *cpsF*. We further identified urease-related genes *ureB* and *ureG* (which are similar to those of *Helicobacter*) and the *tviB* antigen; B91_SC and B91_TPP strains contained RNA polymerase factor sigma-54, which plays a role in regulation of *flaB* expression and host colonization in *C. jejuni* [42].

For antibiotic resistance, we identified inner membrane transporters AcrF and MexQ*,* as well as the membrane fusion protein CmeA in all strains. MacA and MacB were encoded by all strains, except that the latter was not encoded by CC57c. Together, these two proteins form an antibiotic efflux complex with TolC*,* which was not present in any strain [56] (Supplementary Figure 5). No plasmid-borne antibiotic resistance genes were identified. Overall, antibiotic resistance genes were rare and difficult to detect, and *C. showae* only contains a subset of the virulence genes present in *C. jejuni*.

### Gene families within gut-derived *C. showae* isolates segregate with oral metagenomes, based on location

*C. showae* is indigenous to the human oral cavity, but all strains isolated in this study were from the colon or stool (Table 1). To better understand how the colonic *C. showae* strains may differ from oral strains, we compared gene family presence/absence in our *C. showae* genomes to *C. showae* in metagenomes from the expanded Human Microbiome Project (HMP1-II) [27]. To do this, we examined HUMAnN2 (http://huttenhower.sph.harvard.edu/humann2) gene family profiles for HMP1-II metagenomes where *C. showae* recruited reads to (1) ≥500 UniRef90 gene families, with (2) reasonably strong and consistent coverage (see Methods). This included 64 oral metagenomes in which *C. showae* contained on average 0.495%, 0.662%, and 0.468% abundances in supragingival plaque (*n* = 43), tongue dorsum (*n* = 18), and buccal mucosa (*n* = 3), respectively. Unfortunately, this filtered out all gut metagenomes, but included 64 oral metagenomes in which C. showae contained on average 0.495%, 0.662%, and 0.468% abundances in supragingival plaque (*n* = 43), tongue dorsum (*n* = 18), and buccal mucosa (*n* = 3), respectively. To make the isolate genomes more comparable to the metagenomes, we aligned each isolate gene to HUMAnN2 *C. showae-*specific gene families and annotated them with the top match. A total of 3 981 UniRef90 gene families were present across all metagenomes and genomes, and 1 936 had non-trivial annotations. 123 were found only in the metagenomes, with 27 found exclusively in the genomes (Supplemental Figure 7).

We first reviewed whether protein families that were differential between adherent/invasive and non-adherent/non-invasive strains were also found in the oral metagenomes. We were unable to detect filamentous hemagglutinin with this method of gene annotation, but observed that the T4SS, S-layer proteins, the hemoglobin and hemoglobin-haptoglobin-binding protein B (identified via VFDB), and CRISPR proteins were present across a majority of tongue dorsum and supragingival plaque metagenomes (Supplemental Table 13). The RTX protein and four hemolysin-type calcium binding domain proteins were unique to CC57c and relatively rare across oral metagenomes. The former was present in 2 tongue dorsum and 15 supragingival plaque metagenomes, while the latter four were found in only 0–2 tongue dorsum and 1–13 supragingival plaque metagenomes. In contrast, a putative hemolysin was found in CC57c, CAM, the three strains from patient B91, in all 43 supragingival plaque metagenomes and in 11 of 18 tongue dorsum metagenomes.

**Figure 5.**
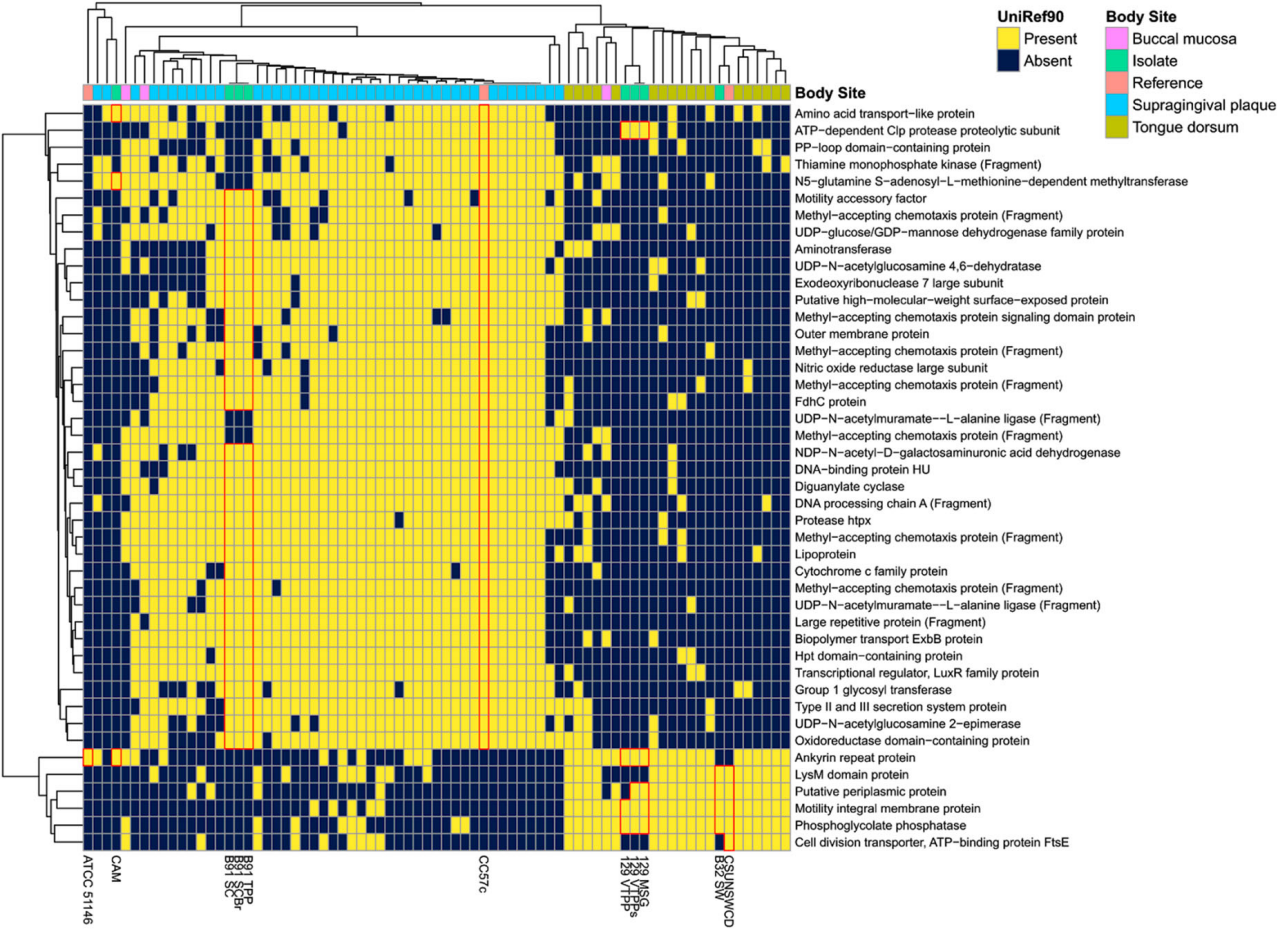
*C. showae* gut isolate strains differ in their functional capacity from strains observed in oral metagenomes. Data show gene families with informative UniRef90 annotations were contained in this study’s isolates or in at least 75% of supragingival plaque metagenomes and less than 25% in tongue dorsum metagenomes from the Human Microbiome Project (or vice-versa). Clustering is by Jaccard distance.

We next looked to see whether colonic strains might preferentially share genes with *C. showae* metagenomes from the tongue dorsum, supragingival plaque, or neither body site (Supplemental Figure 7). Strains from patient 129, B32_SW, and CSUNSWCD contained multiple genes seen in a majority of tongue dorsum metagenomes and a minority of supragingival plaque metagenomes while strains from patients B91 and CC57c carried genes with the opposite pattern (Figure 5). The former contained genes for a periplasmic protein, motility membrane protein, and phosphoglycolate phosphatase while the latter had several methyl-accepting chemotaxis proteins, UDP-N-acetylglucosamine enzymes, and Type II and III secretion system proteins. Lastly, the prepilin-type cleavage/methylation N-terminal proteins were prevalent across oral metagenomes and present only in CC57c and in oral strain ATCC 51146. Genes with this pattern may be specific to oral strains and eventually lost in strains that adapt to persistence (or pathogenicity) in the gut. Prepilin-like proteins are often used to form type IV pili and biofilms, and they are also necessary for enzyme and toxin secretion [57].

## Discussion and conclusion

Here, a combination of experimental characterization and comparative genomics shows that the emerging intestinal pathogenic species *Campylobacter showae* exhibits a range of phenotypes explained by differences in the presence/absence of variety of gene families. Notably, strains from one patient (129) are adherent/invasive, whereas those from patient B91 are not. This may result from their different hosts and diagnoses; the former is from an adult patient with colonic adenoma, while the latter is derived from a pediatric patient with Crohn’s disease. The B32_SW strain is also from a pediatric patient with Crohn’s disease, yet shows more phenotypic and genomic similarity to the strains from patient 129. This suggests that the pangenomic diversity of *C. showae* can have clinical consequences for its hosts, potentially necessitating more careful management of infections. Some observed gene differences, such as that of the T4SS, clearly segregates with adherent/invasive phenotypes, while genes such as methyl-accepting chemotaxis proteins are primarily found in supragingival plaque metagenomes. Still, the majority of gene families have no meaningful annotation or match with databases VFDB and CARD, indicating that the current mechanistic characterization of *C. showae* remains incomplete. By combining functional assessments with genome comparisons, our study significantly adds to the current knowledge base and contributes to our understanding of the evolution and adaptation of bacterial virulence of *C. showae* in the gut environment.

## Supplementary Material

Supplemental Material

## Sequence deposition

The *C. concisus* raw sequencing reads and genome assemblies are freely available from the EMBL-EBI ENA under the study Accession PRJEB17707.
