# Supplementary material for: Comparative genomics and genome biology of Campylobacter showae
Source: Emerg Microbes Infect. 2019 Jun 6;8(1):827–40. doi: 10.1080/22221751.2019.1622455 (PMC6567213; doi:10.1080/22221751.2019.1622455)
Supplement: Supplemental Material [file TEMI_A_1622455_SM4626.zip › Supplementary Material/Supplementary table 13.zip!/Supplementary_Table_1.docx]

# **Supplementary Table 12: Real-time PCR primers and efficiencies used in this study.**

| Gene | Primer sequences 5’ - 3’ | Assay efficiency (%) | Product size (bp) |
| --- | --- | --- | --- |
| glucose-6-isomerase | Forward 5’-AGCTTGAGCGGCTAGATGAG  Reverse 5’-GGCCGTTAGCATCGTCTTTA | 96.4 | 152 |
| PomA | Forward 5’-GATGCCGTAAAACGTGCCTA  Reverse 5’-TCGGAATCGGAGCTTACATC | 96.6 | 122 |
| TonB | Forward 5’-TGGGCTTATAGTGGCGATTC  Reverse 5’-ACAGGCTCATAATGCGGTTT | 93.5 | 98 |
| HopAH2-2 | Forward 5’-GGACGATGATCTCGTTTTGC  Reverse 5’-CAACTCGGGCAAATGTAAGG | 93.3 | 140 |
| HlyD | Forward 5’-AAAGTGGCTCACCCTCTTGA  Reverse 5’-GGCTACGTCGAGGGAGATTA | 98.6 | 106 |
| AccA | Forward 5’-GTCATCCGGATCGTCCTTA  Reverse 5’-CGCCTATCACGACGACTTTT | 97.7 | 150 |
| FlaB | Forward 5’-TACGCGGTGCGATGG  Reverse 5’-CTGACTCGGCAGCGAAA | 95.2 | 186 |
