# Supplementary material for: Comparative genomics and genome biology of Campylobacter showae
Source: Emerg Microbes Infect. 2019 Jun 6;8(1):827–40. doi: 10.1080/22221751.2019.1622455 (PMC6567213; doi:10.1080/22221751.2019.1622455)
Supplement: Supplemental Material [file TEMI_A_1622455_SM4626.zip › Supplementary Material/Supplementary table 13.zip!/Supplementary_Table_10.docx]

# **Supplementary Table 10: Plasmid assembly and annotation statistics.**

|  | B32_SW | B91_SC | B91_SCBr | B91_TPP | CAM | 129_MSG | 129_VTPP | 129_VTPPs | CC57c | CSUNSWCD |
| --- | --- | --- | --- | --- | --- | --- | --- | --- | --- | --- |
| Number assembled Plasmids | 3 | 4 | 1 | 4 | 15 | 1 | 19 | 2 | 3 | 8 |
| Total Length of Plasmids | 42,349 | 43,994 | 19196 | 57,856 | 522,387 | 29677 | 154,136 | 56622 | 45534 | 79596 |
| N50 of plasmids | 31,086 | 15,109 | 19196 | 16,144 | 62,112 | 29677 | 17406 | 29803 | 31422 | 41962 |
| **Prokka** | | | | | | | | | | |
| CDS | 61 | 40 | 19 | 51 | 513 | 30 | 161 | 42 | 50 | 118 |
| Gene | 61 | 41 | 19 | 52 | 525 | 30 | 161 | 42 | 50 | 118 |
| repeat region | 0 | 0 | 0 | 0 | 0 | 0 | 0 | 0 | 0 | 0 |
| tRNA | 0 | 1 | 0 | 1 | 12 | 0 | 0 | 0 | 0 | 0 |
| **CARD** | | | | | | | | | | |
| CARD AROs identified | 51 | 34 | 14 | 41 | 452 | 28 | 135 | 42 | 49 | 102 |
| Strict criteria | 0 | 0 | 0 | 0 | 0 | 0 | 0 | 0 | 0 | 0 |
| Loose criteria | 51 | 34 | 14 | 41 | 452 | 28 | 135 | 42 | 49 | 102 |
| # prot hits (bitscore > 50) | 0 | 4 | 2 | 4 | 33 | 0 | 3 | 2 | 0 | 0 |
| # prot hits (80% cov cutoff) | 0 | 3 | 1 | 3 | 21 | 0 | 1 | 2 | 0 | 0 |
| **VFDB** | | | | | | | | | | |
| # prot hits (bitscore > 50) | 6 | 9 | 6 | 10 | 65 | 8 | 22 | 11 | 6 | 0 |
| # prot hits (80% cov cutoff) | 5 | 1 | 0 | 1 | 16 | 0 | 6 | 1 | 5 | 0 |
