# Supplementary material for: Comparative genomics and genome biology of Campylobacter showae
Source: Emerg Microbes Infect. 2019 Jun 6;8(1):827–40. doi: 10.1080/22221751.2019.1622455 (PMC6567213; doi:10.1080/22221751.2019.1622455)
Supplement: Supplemental Material [file TEMI_A_1622455_SM4626.zip › Supplementary Material/Supplementary table 13.zip!/Supplementary_table_11.docx]

# **Supplementary Table 11: Plasmid to Plasmid blast statistics.** Blast results only displayed for hits where the subject and query percentage alignment were above 50%.

| query ID | subject ID | query Plasmid | subject Plasmid | %age  Identity | query  Length | subject  Length | aln  Length | mismatch  Count | gap  Open  Count | query  Start | query  End | subject  Start | subject  End | e-Value | bit  Score | query  %age  Alignment | subject  %age  Alignment |
| --- | --- | --- | --- | --- | --- | --- | --- | --- | --- | --- | --- | --- | --- | --- | --- | --- | --- |
| B32_SW | 129_VTPP | 1 | 3 | 99.51 | 31087 | 22790 | 21483 | 100 | 4 | 8050 | 29530 | 1 | 21480 | 0 | 39085 | 69.11 | 94.27 |
| B32_SW | CC57c | 1 | 1 | 98.53 | 31087 | 31423 | 22260 | 312 | 5 | 8839 | 31086 | 31422 | 9167 | 0 | 39275 | 71.61 | 70.84 |
| B32_SW | CSUNSWCD | 2 | 3 | 95.84 | 9820 | 10158 | 5795 | 229 | 12 | 4031 | 9819 | 6229 | 441 | 0 | 9356 | 59.01 | 57.05 |
| B91_SC | B91_SCBr | 1 | 1 | 100 | 17210 | 19197 | 17209 | 0 | 0 | 1 | 17209 | 19195 | 1987 | 0 | 31780 | 99.99 | 89.64 |
| B91_SC | B91_TPP | 1 | 1 | 100 | 17210 | 16867 | 16863 | 0 | 0 | 1 | 16863 | 16863 | 1 | 0 | 31141 | 97.98 | 99.98 |
| B91_SC | B91_TPP | 2 | 2 | 100 | 15110 | 16145 | 15109 | 0 | 0 | 1 | 15109 | 15209 | 101 | 0 | 27902 | 99.99 | 93.58 |
| B91_SC | B91_TPP | 3 | 3 | 100 | 10133 | 12506 | 10132 | 0 | 0 | 1 | 10132 | 20 | 10151 | 0 | 18711 | 99.99 | 81.02 |
| B91_SCBr | B91_TPP | 1 | 1 | 100 | 19197 | 16867 | 16864 | 0 | 0 | 2333 | 19196 | 1 | 16864 | 0 | 31143 | 87.85 | 99.98 |
| 129_MSG | 129_VTPP | 1 | 1 | 100 | 29678 | 25931 | 25919 | 0 | 0 | 3759 | 29677 | 1 | 25919 | 0 | 47864 | 87.33 | 99.95 |
| 129_MSG | 129_VTPPs | 1 | 1 | 100 | 29678 | 29804 | 29676 | 0 | 0 | 2 | 29677 | 118 | 29793 | 0 | 54802 | 99.99 | 99.57 |
| 129_VTPP | 129_VTPPs | 1 | 1 | 100 | 25931 | 29804 | 25928 | 0 | 0 | 1 | 25928 | 3875 | 29802 | 0 | 47881 | 99.99 | 87.00 |
| 129_VTPP | CC57c | 3 | 1 | 98.82 | 22790 | 31423 | 20691 | 242 | 2 | 790 | 21480 | 31422 | 10734 | 0 | 36856 | 90.79 | 65.85 |
| 129_VTPPs | 129_VTPP | 1 | 1 | 100 | 29804 | 25931 | 25928 | 0 | 0 | 3875 | 29802 | 1 | 25928 | 0 | 47881 | 87.00 | 99.99 |
| CSUNSWCD | CSUNSWCD | 5 | 6 | 83.12 | 2033 | 1012 | 1031 | 149 | 15 | 1 | 1026 | 1 | 1011 | 0 | 917 | 50.71 | 101.88 |
