# Supplementary material for: Comparative genomics and genome biology of Campylobacter showae
Source: Emerg Microbes Infect. 2019 Jun 6;8(1):827–40. doi: 10.1080/22221751.2019.1622455 (PMC6567213; doi:10.1080/22221751.2019.1622455)
Supplement: Supplemental Material [file TEMI_A_1622455_SM4626.zip › Supplementary Material/Supplementary table 13.zip!/Supplementary_Table_2.docx]

# **Supplementary Table 2: BUSCO results.** Assessment of the genome completeness with the use of Benchmarking Universal Single-Copy Orthologs (BUSCOs). BUSCOs were searched for in the genome fasta files created by PROKKA. The Delta+Epsilonproteobacteria BUSCO DB was used.

|  | B32_SW | B91_SC | B91_SCBr | B91_TPP | CAM | 129_MSG | 129_VTPP | 129_VTPPs | CC57c | ATCC 51146 | CSUNSWCD |
| --- | --- | --- | --- | --- | --- | --- | --- | --- | --- | --- | --- |
| % Complete | 98.3 | 98.0 | 98.0 | 98.0 | 98.3 | 98.0 | 98.3 | 98.3 | 92.9 | 98.3 | 97.6 |
| Complete BUSCOs | 291 | 290 | 290 | 290 | 291 | 290 | 291 | 291 | 275 | 291 | 289 |
| Complete and single-copy BUSCOs | 290 | 289 | 289 | 289 | 290 | 289 | 290 | 290 | 275 | 290 | 288 |
| Complete and duplicated BUSCOs | 1 | 1 | 1 | 1 | 1 | 1 | 1 | 1 | 0 | 1 | 1 |
| Fragmented BUSCOs | 1 | 2 | 2 | 2 | 2 | 1 | 1 | 1 | 12 | 1 | 1 |
| Missing BUSCOs | 4 | 4 | 4 | 4 | 3 | 5 | 4 | 4 | 9 | 4 | 6 |
| Total BUSCO groups searched | 296 | 296 | 296 | 296 | 296 | 296 | 296 | 296 | 296 | 296 | 296 |
