# Supplementary material for: Comparative genomics and genome biology of Campylobacter showae
Source: Emerg Microbes Infect. 2019 Jun 6;8(1):827–40. doi: 10.1080/22221751.2019.1622455 (PMC6567213; doi:10.1080/22221751.2019.1622455)
Supplement: Supplemental Material [file TEMI_A_1622455_SM4626.zip › Supplementary Material/Supplementary table 13.zip!/Supplementary_Table_3.docx]

# **Supplementary Table 3: REAPR genome assembly evaluation.** Assessment of errors within genome assemblies. FCD errors refers to Fragment Coverage Distribution Errors based on the difference between the theoretical and observed FCD. FCD errors and low fragment coverage refer to regions that do not contain a gap. Results for CAM are unavailable due to good quality Illumina sequencing not being available.

| **Sample** | **B32_SW** | **B91_SC** | **B91_SCBr** | **B91_TPP** | **CAM** | **129_MSG** | **129_VTPP** | **129_VTPPs** |
| --- | --- | --- | --- | --- | --- | --- | --- | --- |
| **bases** | 2308370 | 2160580 | 2138501 | 2149118 | NA | 2204388 | 2592634 | 2240249 |
| **error free** | 1993707 | 1716319 | 1964629 | 2029785 | NA | 1949156 | 1649394 | 2046876 |
| **% error free** | 86.36 | 79.44 | 91.87 | 94.45 | NA | 88.42 | 63.62 | 91.37 |
| **gaps** | 0 | 0 | 31 | 32 | NA | 0 | 51 | 26 |
| **gaps bases** | 0 | 0 | 14074 | 12049 | NA | 0 | 22551 | 13444 |
| **FCD errors** | 1 | 1 | 4 | 15 | NA | 10 | 4 | 3 |
| **FCD errors over a gap** | 0 | 0 | 0 | 0 | NA | 0 | 1 | 0 |
| **Low fragment coverage** | 5427 | 5970 | 4240 | 1308 | NA | 4325 | 4558 | 3992 |
| **Low fragment coverage over a gap** | 0 | 0 | 20 | 20 | NA | 0 | 26 | 8 |
