# Supplementary material for: Comparative genomics and genome biology of Campylobacter showae
Source: Emerg Microbes Infect. 2019 Jun 6;8(1):827–40. doi: 10.1080/22221751.2019.1622455 (PMC6567213; doi:10.1080/22221751.2019.1622455)
Supplement: Supplemental Material [file TEMI_A_1622455_SM4626.zip › Supplementary Material/Supplementary table 13.zip!/Supplementary_Table_4.docx]

# **Supplementary Table 4: Genome statistics.** Quast results were calculated on contigs >= 500bp except where stated. The Illumina and PacBio coverage were calculated by aligning the Illumina MiSeq raw paired reads and the PacBio-RSII filtered subreads respectively to the final assembly.

|  | **B32_SW** | **B91_SC** | **B91_SCBr** | **B91_TPP** | **CAM** | **129_MSG** | **129_VTPP** | **129_VTPPs** | **CC57c** | **ATCC 51146** | **CSUNSWCD** |
| --- | --- | --- | --- | --- | --- | --- | --- | --- | --- | --- | --- |
| **Genome Source** | This study | This study | This study | This study | This study | This study | This study | This study | Warren 2013 | Published | Tay et al 2013 |
| **Biological Source*** | CB | CB | CB | CB | F | CB | CB | CB | CT | OGC | CB |
| **Individual Source Condition+** | pCroP | pCroP | pCroP | pCroP | aAGastroP | aAdeP | aAdeP | aAdeP | adult CRC patient | adult periodontitis | pCroP |
| **Sequencing technology^** | IM + PB-RSII | IM + PB-RSII | IM | IM | PB-RSII | IM + PB-RSII | IM | IM | IM | 454 + IH | IH |
| **Illumina Coverage** | 87 | 69 | 104 | 75 | N/A | 38 | 76 | 59 | N/A | N/A | N/A |
| **PacBio Coverage** | 497 | 525 | N/A | N/A | 817 | 708 | N/A | N/A | N/A | N/A | N/A |
| **Quast** | | | | | | | | | | | |
| **# contigs**  **(>= 0 bp)** | 2 | 1 | 4 | 6 | 3 | 4 | 2 | 2 | 274 | 1 | 23 |
| **Total length**  **(>= 0 bp)** | 2,308,370 | 2,160,580 | 2,138,501 | 2,149,118 | 2,502,981 | 2,243,050 | 2,592,634 | 2,240,249 | 2,192,767 | 2,097,888 | 2,125,173 |
| **Largest contig** | 2,157,093 | 2,160,580 | 1586030 | 2,131,013 | 2,162,358 | 1295828 | 1,697,095 | 2,166,083 | 65,929 | 2,097,888 | 401,824 |
| **Total length** | 2,308,370 | 2,160,580 | 2138501 | 2,149,118 | 2,502,981 | 2243050 | 2,592,634 | 2,240,249 | 2,182,115 | 2,097,888 | 2,124,667 |
| **GC (%)** | 44 | 46 | 46 | 46 | 44 | 45 | 45 | 45 | 45 | 46 | 45 |
| **N50** | 2,157,093 | 2,160,580 | 1586030 | 2,131,013 | 2,162,358 | 1295828 | 1,697,095 | 2,166,083 | 16,877 | 2,097,888 | 209,023 |
| **N75** | 2,157,093 | 2,160,580 | 359,805 | 2,131,013 | 2,162,358 | 633,172 | 895,539 | 2,166,083 | 8,593 | 2,097,888 | 110,105 |
| **L50** | 1 | 1 | 1 | 1 | 1 | 1 | 1 | 1 | 42 | 1 | 4 |
| **L75** | 1 | 1 | 2 | 1 | 1 | 2 | 2 | 1 | 88 | 1 | 8 |
| **ENA accession numbers (ENA Accession = PRJEB17707)** | | | | | | | | | | | |
| **Genome assemblies** | GCA_900573925.1 | LR535679.1 | GCA_900573945.1 | GCA_900573955.1 | GCA_900573985.1 | GCA_900573995.1 | GCA_900573955.1 | GCA_900573975.1 | N/A | N/A | N/A |
| **Illumina Sample Accession** | SAMEA4535846 | SAMEA4535847 | SAMEA4535848 | SAMEA4535849 | SAMEA4535854 | SAMEA4535850 | SAMEA4535851 | SAMEA4535852 | N/A | N/A | N/A |
| **PacBio Sample Accession** | SAMEA4535862 | SAMEA4535863 | N/A | N/A | SAMEA4535865 | SAMEA4535864 | N/A | N/A | N/A | N/A | N/A |

* CB depict Colonic biopsy, CT depicts Colonic tissue, OGC depicts Oral gingival crevice, F depicts faecal sample

+ pCroP depicts paediatric Crohn's patient, aAdeP depicts adult adenoma patient, aAGastroP depicts adult acute gastroenteritis patient

^ IM depicts Illumina MiSeq, IH depicts Illumina HiSeq, 454 depicts 454 Titanium and PB-RSII depicts PacBio-RSII

Warren et al 2013, PMID: 24450771;Tay et al 2013, PMID: 2340925
