# Supplementary material for: Comparative genomics and genome biology of Campylobacter showae
Source: Emerg Microbes Infect. 2019 Jun 6;8(1):827–40. doi: 10.1080/22221751.2019.1622455 (PMC6567213; doi:10.1080/22221751.2019.1622455)
Supplement: Supplemental Material [file TEMI_A_1622455_SM4626.zip › Supplementary Material/Supplementary table 13.zip!/Supplementary_Table_5.docx]

# **Supplementary Table 5: Genome annotation.** Genome indel assess shows the difference in length of Prodigal annotated proteins against their best hit protein in the Swissprot database. More info in Supplementary methods.

|  | **B32_SW** | **B91_SC** | **B91_SCBr** | **B91_TPP** | **CAM** | **129_MSG** | **129_VTPP** | **129_VTPPs** | **CC57c** | **ATCC 51146** | **CSUNSWCD** | ***C. jejuni* NCTC 11168** |
| --- | --- | --- | --- | --- | --- | --- | --- | --- | --- | --- | --- | --- |
| **Prokka info** | | | | | | | | | | | |  |
| **repeat region** | 0 | 1 | 1 | 1 | 5 | 0 | 0 | 0 | 1 | 1 | 0 | 1 |
| **gene** | 2,351 | 2,117 | 2,096 | 2,096 | 2,449 | 2,273 | 2,627 | 2,268 | 2,214 | 2,055 | 2,174 | 1,703 |
| **CDS** | 2,301 | 2,069 | 2,052 | 2,052 | 2,395 | 2,225 | 2,579 | 2,224 | 2,188 | 2,006 | 2,136 | 1,658 |
| **tRNA** | 49 | 47 | 43 | 43 | 47 | 47 | 46 | 43 | 25 | 48 | 37 | 44 |
| **tmRNA** | 1 | 1 | 1 | 1 | 1 | 1 | 2 | 1 | 1 | 1 | 1 | 1 |
| **Genome Indel Assess** | | | | | | | | | | | | |
| **Proteins (Prodigal annotated)** | 1,184 | 1,200 | 1,186 | 1,194 | 1,243 | 1,201 | 1,389 | 1,201 | 1,239 | 1,150 | 1,153 | 1,124 |
| **Proteins < 0.95** | 115 | 115 | 115 | 121 | 146 | 126 | 147 | 126 | 246 | 97 | 108 | 109 |
| **Proteins >= 0.95 & < 1.05** | 809 | 823 | 810 | 810 | 821 | 806 | 929 | 807 | 722 | 790 | 789 | 803 |
| **Proteins >= 1.05** | 260 | 262 | 261 | 263 | 276 | 269 | 313 | 268 | 271 | 263 | 256 | 212 |
| **CARD** | | | | | | | | | | | |  |
| **CARD AROs identified** | 1967 | 1821 | 1809 | 1801 | 2269 | 1911 | 2263 | 1949 | 1744 | 1908 | 1858 | 1434 |
| **Strict criteria** | 8 | 8 | 6 | 6 | 7 | 7 | 7 | 7 | 7 | 5 | 8 | 10 |
| **Loose criteria** | 1959 | 1813 | 1803 | 1795 | 2262 | 1904 | 2256 | 1942 | 1737 | 1903 | 1850 | 1424 |
| **# prot hits (80% cov cutoff)** | 8 | 8 | 8 | 8 | 5 | 8 | 8 | 8 | 9 | 9 | 8 | 66 |
| **VFDB (include loose)** | | | | | | | | | | | |  |
| **# prot hits (80% cov cutoff)** | 91 | 86 | 86 | 86 | 95 | 91 | 91 | 91 | 88 | 84 | 90 | 144 |
