# Supplementary material for: Comparative genomics and genome biology of Campylobacter showae
Source: Emerg Microbes Infect. 2019 Jun 6;8(1):827–40. doi: 10.1080/22221751.2019.1622455 (PMC6567213; doi:10.1080/22221751.2019.1622455)
Supplement: Supplemental Material [file TEMI_A_1622455_SM4626.zip › Supplementary Material/Supplementary table 13.zip!/Supplementary_Table_6.docx]

# **Supplementary Table 6: Classification of B91 strains with the use of CLARK.** B91 strain genomes were compared against genomes for the *Campylobacter* species - *coli, concisus, corcagienisis, cuniculorum, curvus, fetus, gracilis, hepaticus, hominis, iguaniorum, insulaenigrae, jejuni, lari, mucosalis, peloridis, rectus, sputorum, subantarcticus, showae, upsaliensis, ureolyticus, volucris*. More information on the genomes in the methods. Classification of NA indicates that CLARK was unable to classify the scaffold.

| **Isolate** | **Scaffold** | **length (bp)** | **Clark classification** |
| --- | --- | --- | --- |
| B91_SC | scaffold1 | 2160580 | *C. showae* |
| B91_SCBr | scaffold1 | 1586030 | *C. showae* |
|  | scaffold2 | 359805 | *C. showae* |
|  | scaffold3 | 1611 | NA |
|  | scaffold4 | 191055 | *C. showae* |
|  | scaffold5 | 19196 | *C. showae* |
| B91_TPP | scaffold1 | 2131013 | *C. showae* |
|  | scaffold2 | 14986 | *C. showae* |
|  | scaffold3 | 916 | NA |
|  | scaffold4 | 847 | NA |
|  | scaffold5 | 751 | *C. concisus* |
|  | scaffold6 | 605 | NA |
