# Supplementary material for: Comparative genomics and genome biology of Campylobacter showae
Source: Emerg Microbes Infect. 2019 Jun 6;8(1):827–40. doi: 10.1080/22221751.2019.1622455 (PMC6567213; doi:10.1080/22221751.2019.1622455)
Supplement: Supplemental Material [file TEMI_A_1622455_SM4626.zip › Supplementary Material/Supplementary table 13.zip!/Supplementary_Table_7.docx]

# **Supplementary Table 7: Detected SNPs & recombination with the C. showae strain genomes.** The number of SNPs and recombinations were detected with Gubbins using ATCC 51146 as a reference.

| Strain | Total SNPs | Num of SNPs inside recombinations | Num of SNPs outside recombinations | Num of Recombination Blocks | Bases in Recombinations |
| --- | --- | --- | --- | --- | --- |
| 129_MSG | 11 | 0 | 11 | 0 | 74,770 |
| 129_VTPP | 45 | 14 | 31 | 2 | 78,870 |
| 129_VTPPs | 0 | 0 | 0 | 0 | 78,938 |
| B32_SW | 15,846 | 10,681 | 5,165 | 225 | 556,796 |
| B91_SC | 62 | 17 | 45 | 2 | 4,584 |
| B91_SCBr | 19 | 5 | 14 | 1 | 4,553 |
| B91_TPP | 0 | 0 | 0 | 0 | 4,550 |
| CAM | 0 | 0 | 0 | 0 | 4,388 |
| CC57c | 60,765 | 723 | 60,042 | 26 | 15,975 |
| CSUNSWCD | 12,686 | 7,307 | 5,379 | 170 | 513,351 |
| ATCC 51146 | 6,068 | 138 | 5,930 | 5 | 10,961 |
