# Supplementary material for: Comparative genomics and genome biology of Campylobacter showae
Source: Emerg Microbes Infect. 2019 Jun 6;8(1):827–40. doi: 10.1080/22221751.2019.1622455 (PMC6567213; doi:10.1080/22221751.2019.1622455)
Supplement: Supplemental Material [file TEMI_A_1622455_SM4626.zip › Supplementary Material/Supplementary table 13.zip!/Supplementary_Table_9.docx]

# **Supplementary Table 9: Identified Genomic Islands within *C. showae* isolates**

| **Strain** | **Region name** | **Approximate start site*** | | **No. of genes** | **Mobility genes present** |
| --- | --- | --- | --- | --- | --- |
|  |  | **Start** | **End** |  |  |
| B32_SW | B32_SW1  B32_SW2  B32_SW3  B32_SW4 | 289,699  548,892  2,158,332  2,226,566 | 324,599  570,045  2,194,210  2,299,254 | 36  23  34  76 | Transposases and Inteferase  Recombinases  Recombinase  Helicase |
| B91_SC | B91_SC1  B91_SC2  B91_SC3  B91_SC4  B91_SC5 | 483,986  508,731  990,614  1,061,321  1,594,287 | 499,190  515,077  1,036,719  1,066,668  1,620,831 | 21  12  46  6  21 | Prophage tail protein  None  Hemolysin secretion  None  Membrane anchor proteins |
| B91_SCBr | B91_SCBr1  B91_SCBr2  B91_SCBr3  B91_SCBr4  B91_SCBr5 | 23,734  86,419  1,158,634  1,812,803  1,837,548 | 61,817  91,766  1,186,586  1,828,007  1,843,894 | 37  6  20  21  12 | Hemolysin secretion  None  Membrane anchor proteins  Prophage tail protein  None |
| B91_TPP | B91_TPP1  B91_TPP2  B91_TPP3  B91_TPP4  B91_TPP5 | 220,947  1,254,472  1,279,217  1,769,728  1,832,387 | 249,508  1,269,676  1,285,563  1,807,785  1,837,734 | 21  21  12  37  6 | Membrane anchor proteins  Prophage tail protein  None  Hemolysin secretion  None |
| 129_MSG | 129_MSG1  129_MSG2  129_MSG3  129_MSG4  129_MSG5 | 31,310  77,281  284,562  2,094,341  2,205,247 | 43,924  89,166  291,690  2,160,797  2,239,337 | 14  10  6  57  34 | Recombinase  None  None  Integrase  None |
| 129_VTPP | 129_VTPP1  129_VTPP2  129_VTPP3  129_VTPP4 | 1,249  430,593  2,298,225  2,345,417 | 36,835  492,875  2,305,383  2,357,986 | 34  51  6  14 | None  Integrase  None  None |
| 129_VTPPs | 129_VTPPs1  129_VTPPs2  129_VTPPs3  129_VTPPs4  129_VTPPs5  129_VTPPs6 | 532,731  579,893  625,638  874,932  917,020  2,207,128 | 539,859  592,507  637,523  908,995  941,734  2,237,890 | 6  14  10  30  27  34 | None  Recombinase  None  Integrase  Integrase  None |
| CAM | CAM1  CAM2 | 20,821  1,261,294 | 283,255  1,287,419 | 234  30 | CRISPR associated proteins  None |
| ATCC51146** | 51146_1  51146_2 | 873,846  1,681,465 | 888,390  1,709,912 | 16  30 | Flagellar hook protein  Integrase |
| CSUNSWCD** | CSUNSWCD1  CSUNSWCD2 | 116,178  1,033,045 | 145,324  1,045,216 | 29  7 | Integrase, Transferase  None |
| CC57c** | CC57c1  CC57c2  CC57c3  CC57c4  CC57c5 | 1,728,888  1,896,676  2,204,269  2,316,374  2,367,650 | 1,737,148  1,919,191  2,284,402  2,352,167  2,404,927 | 9  15  39  20  18 | None  Helicase  Methyl-accepting chemotaxis protein, Transferase  Transferase, Integrase  Hemolysin secretion |

* Whole genome length was estimated using Mauve to order the contigs

** No PCR validation was undertaken
